# Improved Survival in Malnourished COVID-19 Inpatients with Oral Nutrition Supplementation

**DOI:** 10.3390/nu17152401

**Published:** 2025-07-23

**Authors:** Tyrus Vong, Lisa R. Yanek, Laura E. Matarese, Berkeley N. Limketkai, Gerard E. Mullin

**Affiliations:** 1 Department of Medicine, Division of Gastroenterology and Hepatology, Johns Hopkins University School of Medicine, Baltimore, MD 21287, USA; tvong2@jhmi.edu; 2 Department of Medicine, Division of General Internal Medicine, Johns Hopkins University School of Medicine, Baltimore, MD 21287, USA; lryanek@jhmi.edu; 3 Department of Internal Medicine, Division of Gastroenterology, Hepatology, and Nutrition, East Carolina University, Greenville, NC 27834, USA; mataresel@ecu.edu; 4 Division of Digestive Diseases, UCLA School of Medicine, Los Angeles, CA 90095, USA

**Keywords:** COVID-19, SARS-CoV-2, nutrition, malnutrition, oral nutrition supplementation, mortality, survival

## Abstract

Background: Malnutrition is associated with adverse clinical and economic outcomes. We recently reported that the hospital mortality rate in severe acute respiratory syndrome coronavirus 2 (SARS-CoV-2)-infected inpatients was higher in malnourished patients than in those without malnutrition. The present study aimed to determine if SARS-CoV-2-infected inpatients who received oral nutrition supplementation (ONS) had improved survival. We performed a retrospective cohort study including 37,215 adults (aged 18 and older) admitted with COVID-19 to five Johns Hopkins–affiliated hospitals between 1 March 2020, and 31 March 2023. Malnutrition risk was initially screened using the Malnutrition Universal Screening Tool (MUST), with cases subsequently confirmed by registered dietitians via a standardized, validated assessment protocol. Logistic regression analysis predicting hospital mortality examined the association of ONS with hospital survival in SARS-CoV-2-infected inpatients, incorporating covariates and weights for ONS receipt. Results: Malnutrition was an independent predictor of higher hospital mortality from COVID-19 illness. The prevalence of malnutrition among adult inpatients with SARS-CoV-2 infection in our cohort was 15.22%. Inpatient adults with moderate or severe malnutrition in the context of acute illness or injury who were given ONS had lower odds of inpatient mortality (moderate OR = 0.72, 95% CI 0.62–0.85; severe OR = 0.76, 95% CI 0.67–0.87; both *p* < 0.001). Overweight and obese patients who received ONS had higher odds of inpatient mortality (overweight OR = 1.15, 95% CI 1.08–1.22, *p* < 0.0001; obese OR = 1.08, 95% CI 1.01–1.14, *p* = 0.02, respectively). For inpatients who were underweight, receiving ONS was protective against inpatient mortality (OR = 0.78, 95% CI 0.68–0.88, *p* = 0.0001). Thus, among adult inpatients with SARS-CoV-2 infection, malnourished and underweight individuals appeared to experience improved survival when provided with oral nutritional supplements (ONS), whereas overweight or obese patients remain at an elevated risk of mortality. The timing of ONS receipt in hospitalized patients with SARS-CoV-2 influenced mortality. Patients who had earlier time to ONS had 13% lower odds of inpatient mortality (OR = 0.87, 95% CI 0.79–0.97, *p* = 0.0105). Conclusions: In a cohort of SARS-CoV-2 adult inpatients, those with confirmed malnutrition receiving oral nutrition supplements had a higher likelihood of hospital survival. This is the first study demonstrating an association of oral nutrition intervention with reduced hospital mortality in malnourished SARS-CoV-2-infected adults.

## 1. Introduction

Severe acute respiratory syndrome coronavirus 2 (SARS-CoV-2), the causative agent of the coronavirus disease 2019 (COVID-19) pandemic, has led to widespread global disease burden. The World Health Organization (WHO) estimates that, from the beginning of the pandemic through 2 February 2025, there have been in excess of 777 million cases and 7 million deaths attributable to SARS-CoV-2 infection [1]. Despite vaccine availability and medications to attenuate its lethal consequences, COVID-19 remains a potentially fatal infectious disease that can quickly lead to cardiopulmonary failure and death [2,3]. Although most of the adults infected by SARS-CoV-2 can be managed in the ambulatory setting, others require hospitalization depending on host cofactors. SARS-CoV-2-infected individuals who are elderly, live in nursing homes, and those with conditions associated with immunocompromise are at higher risk for mortality [4,5]. Comorbidities such as cardiopulmonary disease, diabetes, and obesity have in common chronic systemic inflammation, which is dramatically heightened with SARS-CoV-2 infection. Further, these inflammatory comorbidities compromise lean body mass and often result in malnutrition, which impairs immunity to viral infections such as SARS-CoV-2 [6,7,8,9,10]. Symptomatic infection by SARS-CoV-2 can result in a catabolic inflammatory response that depletes nutritional reserves, further impairs immunity, and amplifies COVID-19 disease severity. Malnutrition in the hospital setting is a well-established risk factor for unfavorable health and economic outcomes [11,12]. Early intervention with oral nutritional supplementation (ONS) in hospitalized patients with cardiopulmonary disease and malnutrition, as demonstrated in a double-blind randomized controlled clinical trial, led to improved clinical and economic outcomes, notably shorter hospital stays and decreased mortality [13]. We previously identified a 9.3% prevalence of malnutrition among 4311 consecutive adult COVID-19 inpatients [14]. Notably, malnourished patients had a significantly higher mortality rate (25.31% [102/403]) compared with their well-nourished counterparts (11.59% [453/3908]; *p* < 0.0001), substantiating malnutrition as a predictor of poor outcomes. Building on these findings, the present study investigates whether oral nutritional supplement (ONS) use is associated with improved survival in hospitalized adults with COVID-19.

## 2. Materials and Methods

### 2.1. Study Design and Participants

We conducted a retrospective cohort study of 37,215 adults (≥18 years) hospitalized with COVID-19 across five Johns Hopkins Medicine (Baltimore, MD, USA) hospitals in Maryland and Washington, DC, from 1 March 2020 to 31 March 2023. Data were obtained from the JH-CROWN PMAP Registry. The study was approved by the Johns Hopkins Medicine Institutional Review Board (IRB00253531) [15]. The Institute for Clinical and Translational Research (ICTR) provided initial data extraction, with subsequent review and approval by the COVID-19 Data Research Evaluation Committee (CADRE). Patients with laboratory-confirmed nasopharyngeal COVID-19 (PCR-positive and ICD-10 code U07.1) were included. Malnutrition was assessed and documented by registered nursing staff [16] and then confirmed by registered dietitians as previously described [14]. Malnutrition was defined by ICD-10 codes E43 or E46 in the electronic health record. Data extraction was completed using Python (version 3.7.5) and included demographics, comorbidities, symptoms, vital signs, medications, laboratory values, and medical history. Initial inpatient admission was designated as the index (time zero) for all analyses. Variables of interest included demographics (sex, age, race, BMI [kg/m^2^]), level of care at admission (medical ward vs. ICU), and comorbidities, which were identified using ICD-10 codes assigned by hospital staff. In-hospital mortality was the primary outcome and was assessed as outlined in the statistical analysis plan below.

### 2.2. Statistical Analysis

#### 2.2.1. Descriptive Analysis

Descriptive statistics were examined, including missingness, means and standard deviations, medians with interquartile ranges, and proportions. Continuous variables were reported as mean ± standard deviation (SD) or as median with interquartile range (IQR), as appropriate. Distributions were assessed, and length of stay and time to ONS variables were log-transformed to normality for analysis. Comparisons between those who received ONS and those who did not receive ONS were tested using Chi-squared tests for categorical variables and *t*-tests for continuous variables. To control for the potential impacts on different “waves” of COVID-19, a ‘COVID-19 wave’ variable was created and incorporated into the multivariate analyses. Waves were defined as pre-vaccine (prior to 31 December 2020), pre-Delta variant (1 January 2021 to 31 May 2021), Delta variant dominant (1 June 2021 to 30 November 2021), Omicron variant dominant (1 December 2021 to 30 April 2022), and post-Omicron (1 May 2022 to 31 March 2023).

#### 2.2.2. Multivariate Analysis

Missingness was assumed to be random. Multiple imputation was applied to all regression analyses to address missing data, utilizing a fully conditional specification approach with ten iterations. The hospital admission date was included as an auxiliary variable to improve imputation accuracy. Inverse probability weighting was used to avoid confounding due to potential selection bias for ONS receipt. Weights were assigned to each patient based on the inverse of their probability of ONS receipt, as estimated by propensity scores calculated from a model including age, sex, race, body mass index, malnutrition diagnosis, date of admission, diabetes, hypertension, diarrhea, chronic obstructive pulmonary disease (COPD), poor appetite, unexplained weight loss, admission source, hospital length of stay and ICU admission. After assuring a balance between groups, multivariable logistic regression analyses incorporating stabilized weights were used to predict inpatient mortality, with additional factors of interest (insurance grouping, COVID wave) incorporated into adjusted models. Models were also run, stratified by malnutrition severity, context, and body mass index category.

Additional models were run only for those patients who received ONS. Multivariable logistic regression analyses were used to predict inpatient mortality, examining time from hospital admission to ONS receipt, including adjustments for age, gender, race, BMI, malnutrition diagnosis, hospital length of stay, ICU admission, diabetes, hypertension, diarrhea, COPD, poor appetite, unexplained weight loss, admission source, COVID wave, and insurance group.

Kaplan–Meier curves were used to visualize time to in-hospital mortality by groups of interest (ONS receipt vs. no ONS receipt, for all patients; patients with malnutrition; stratified by moderate vs. severe malnutrition).

#### 2.2.3. Software

Data analysis was carried out using SAS version 9.4 (SAS Institute Inc., Cary, NC, USA). Statistical significance was determined by a two-sided *p*-value threshold (*p* < 0.05) for all analytic procedures.

## 3. Results

The characteristics of the COVID-19 inpatient cohort are presented in Table 1. A review of these baseline data highlights the distribution of key demographic and clinical features in the study population. A total of 37,215 inpatient patients were analyzed for the study, of which 40.62% were males, 59.36% were females, 0.01% were non-binary, and 0.01% were unknown, with a mean age of 57.56 ± 20.2 years. The patients’ race was 49.68% Whites, 34.51% Black, 4.59% Asian, and 11.22% Other. Of the 37,215 patients admitted, 7.75% expired during their stay. Patients who were readmitted within 30 days were 18.17% were readmitted within 30 days. Patients had a median length of stay in the hospital of 4.1 (2.4–7.6) days and a median length of stay in the ICU of 5.2 (2.8–10) days. The comorbidities of the SARS-CoV-2 inpatients included 15.22% with malnutrition. Of those with malnutrition, 51.64% were severe, and 48.36% were moderate. A subgroup analysis was performed on individuals with available data for malnutrition etiologic context (n = 3628). The context of patients’ malnutrition was 39.66% due to acute disease or injury, 49.97% due to chronic disease or condition, 9.7% due to social or environmental circumstances or starvation, and 0.66% due to other causes. Poor appetite was observed in 13.04% patients, and 7.18% patients reported unintentional weight loss. The patients had a median BMI of 28.3 (24.2–33.5). The patients’ BMI weight categories were 26.52% normal, 40.74% obese, 29.79% overweight, and 2.95% underweight. At discharge, 2.27% patients were ordered ONS, while 97.73% patients were not ordered ONS. During their admission, 20.09% were ordered ONS, and 79.91% were not ordered ONS.

To elucidate, if there was a difference between those who received ONS and those who did not receive ONS during their admission, we stratified the group by ONS order status in Table A1 (Appendix A). The mean age of patients receiving ONS during their admission was higher than the age of those who were not ordered ONS (66.5 ± 17.5) vs. (55.31 ± 20.1), respectively (*p* < 0.0001). The median BMI of patients receiving ONS was lower for those who did not 25.8 (21.9–30.7) vs. 29 (25–34.4), respectively (*p* < 0.0001). Patients given ONS had longer median lengths of stay in the ICU 10.8 (6.3–18.1) days vs. 4.1 (2.3–7.3) days, respectively, (*p* < 0.0001) and in the hospital 10.2 (6.2–17.5) days vs. 3.3 (2.2–5.5) days, respectively, (*p* < 0.0001) than those who were not given ONS. There was a significant difference between groups based on gender, race, insurance group, weight category, and admission source (all *p* < 0.0001). Patients with diabetes, hypertension, diarrhea, COPD, and malnutrition were more likely to receive ONS (all *p* < 0.0001). There was a significant difference between groups based on malnutrition severity, malnutrition context, and COVID wave (*p* = 0.02; *p* < 0.0001; *p* < 0.01, respectively). Patients with poor appetite, unintentional weight loss, and those who had discharge ONS ordered were more likely to receive ONS during their admission (all *p* < 0.0001). Patients who were readmitted to the hospital, admitted to the ICU, or expired during their stay were more likely to receive ONS (all *p* < 0.0001).

Table 2 displays the patient characteristics of those with malnutrition stratified by ONS order status. Of the 5664 patients with malnutrition, 2635 (46.5%) received ONS, while 3029 (53.5%) did not. The mean age at admission was higher in patients given ONS than those not given (ONS 67.17 ± 16.9 vs. 61.77 ± 19.5, respectively, *p* < 0.001). The median BMI of patients receiving ONS was lower than the BMI of those without ONS orders (23.2 (19.9–27.5) vs. 24.9 (20.8–31.6), respectively, *p* < 0.001). The median length of stay in the ICU (12.1 (6.9–22.7)) or hospital (11.1 (6.6–20.8)) for those given ONS was longer than for those not given ONS (ICU 5.3 (2.7–10.9), hospital 4.6 (2.4–8.9), both *p* < 0.0001).

There was a significant difference between groups based on gender, insurance group, weight category, and admission source (*p* = 0.02; *p* < 0.0001 for all others). There was no difference in ONS ordering for patients by race (*p* = 0.52). Patients with hypertension were more likely to receive ONS (*p* < 0.0001). There was no difference in ONS reception patterns for patients with diabetes, diarrhea, or COPD (*p* = 0.53, *p* = 0.28, and *p* = 0.11, respectively). Differences in ONS reception were observed across the three malnutrition context categories and the two malnutrition severities (*p* < 0.0001 and *p* = 0.02, respectively). Patients with poor appetite and unintentional weight loss were more likely to receive ONS (*p* < 0.0001; *p* < 0.0001). Hospital inpatients with SARS-CoV-2 infection and malnutrition were more likely to receive ONS orders at discharge (*p* < 0.0001). ONS provision in hospitalized patients with SARS-CoV-2 differed across the five defined waves of the COVID-19 pandemic (*p* < 0.05). Patients who were readmitted or deceased were more likely to receive ONS (*p* < 0.0001 and *p* < 0.0001, respectively). Patients with malnutrition who were admitted to the ICU were less likely to receive ONS (*p* < 0.0001).

In the overall sample, while ONS receipt was associated with a higher inpatient mortality rate (11.5% vs. 4.6%, *p* < 0.0001) in unadjusted analysis, this effect was attenuated in weighted and adjusted logistic regression analysis (OR = 1.03, 95% CI 0.99–1.06, *p* = 0.1). To elucidate the association(s) between ONS, inpatient mortality, and malnutrition severity and context, multivariable logistic regression analyses were performed and are reported in Table 3. In unadjusted analysis, for patients with malnutrition in the context of acute illness or injury, there was no difference in hospital mortality between inpatients receiving ONS or not, regardless of whether the malnutrition was moderate or severe (*p* = 0.27 and *p* = 0.82, respectively). In unadjusted analyses for patients with malnutrition in the context of chronic illness, inpatient mortality was higher for patients given ONS for both moderate and severe malnutrition (*p* < 0.0001 and *p* = 0.01, respectively). After weighting for receipt of ONS and adjusting for covariates, patients with moderate or severe malnutrition in the context of acute illness or injury who were given ONS have lower odds of inpatient mortality: patients with moderate malnutrition in the context of acute illness or injury who were given ONS were 28% less likely to have inpatient mortality and patients with severe malnutrition in the context of acute illness or injury who were given ONS were 24% less likely to experience inpatient mortality (both *p* < 0.0001). In the case of moderate or severe malnutrition in the context of chronic illness, patients given ONS were still more likely to expire in the weighted and adjusted models, but these effects were slightly attenuated compared to the base model.

Multivariable logistic regression was performed to determine if ONS receipt impacted inpatient mortality for patients with different weight categories, as shown in Table 4. Patients in the normal weight, obese, or overweight categories were significantly more likely to die if they received ONS (*p* < 0.0001), but this was not statistically significant for underweight patients (*p* = 0.0783). In the base model, normal-weight patients who received ONS had 2.43 times higher odds of inpatient mortality than those who did not receive ONS (*p* < 0.0001). ONS was protective of inpatient mortality in the weighted model (OR = 0.947, *p* = 0.0477), but this effect was attenuated in the adjusted model (OR = 0.957, *p* = 0.1148). For obese patients, in the base model, patients with ONS had 3.722 times higher odds of inpatient mortality (*p* < 0.001), although the odds were attenuated to an OR of 1.109 in the weighted model and an OR of 1.075 in the adjusted model (*p* = 0.0008 and *p* = 0.0216, respectively). Overweight patients who received ONS had 3.507 times higher odds of inpatient mortality (*p* < 0.0001), with attenuation in both the weighted (OR = 1.162) and adjusted (OR = 1.148) models (*p* < 0.0001 and *p* < 0.0001, respectively). In the base model, ONS was not significantly associated with inpatient mortality for inpatients who were underweight (*p* = 0.0783). However, ONS was protective against inpatient mortality in the weighted model (OR =0.749, *p* < 0.001) and the adjusted model (OR = 0.776, *p* = 0.0001).

Additional modeling was performed in only those patients who received ONS (n = 7475). Table 5 illustrates the results of a linear regression model examining the relationship between time from hospital admission to receiving ONS and inpatient mortality. Unadjusted analyses showed no difference between patients discharged alive and patients deceased in the hospital in the time from hospital admission to receipt of ONS (*p* = 0.1035). In the adjusted model, patients who had an earlier time to ONS had 13% lower odds of inpatient mortality (*p* = 0.0105).

**Figure 1:**
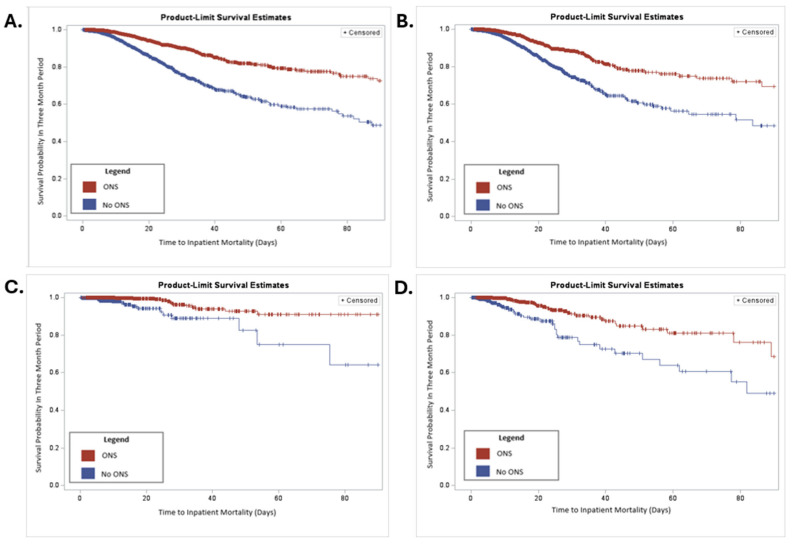
Kaplan–Meier curve of patients treated with or without ONS. (**A**) Kaplan–Meier curve for all patients over 90 days. (**B**) Kaplan–Meier for malnutrition patients over 90 days. (**C**) Kaplan–Meier curve of patients with moderate malnutrition over 90 days. (**D**) Kaplan–Meier curve of patients with severe malnutrition over 90 days. *p* < 0.0001 for all curves.

A series of Kaplan–Meier curves were examined to visualize the projected 90-day survival rates of patients treated with or without ONS, as shown in Figure 1. Patients who received ONS had longer times to inpatient mortality than those who were not given ONS (*p* < 0.001; Figure 1A). When limited to patients who had malnutrition, patients given ONS still had a longer time to inpatient mortality than those who did not receive ONS (*p* < 0.0001; Figure 1B). The same results were seen when stratified by severity of malnutrition. In patients with moderate malnutrition (Figure 1C) and in patients with severe malnutrition (Figure 1D), the estimated time to inpatient mortality was longer for patients who received ONS than for those who did not receive ONS (both *p* < 0.0001).

## 4. Discussion

We analyzed data from 37,215 SARS-CoV-2-infected adult inpatients between 1 March 2020, and 31 March 2023, across our five-hospital health system. Malnutrition was identified in 5664 cases, corresponding to a prevalence of 15.22%. These findings provide valuable insights into the burden of malnutrition among hospitalized COVID-19 patients. We previously reported from the same data source that the prevalence of malnutrition in COVID-19 was 9.3% (403/4311) of hospitalized adult patients in 2020 [14]. In the present study a much larger cohort (37,255) is analyzed over the 5 phases of the pandemic whereby the prevalence of malnutrition was higher (15.22%). Other studies reported higher prevalences of inpatient malnutrition than our study (24.3–52.1%) [17,18,19,20]. However, these studies were limited by a considerably smaller number of patients and exhibited variability in patient characteristics, assessment methods, and institutional practices. Other possible reasons for the disparities in malnutrition prevalence among SARS-CoV-2-infected adult inpatients across these studies may include variability in the severity of illness, age, comorbidities, source of admission, phase of the pandemic studied, and, most importantly, the methodologies used to confirm malnutrition. To our knowledge, this is the most extensive collection of consecutive inpatients assessed for malnutrition over the five distinct phases of the COVID-19 pandemic. Thus, our study likely reflects the true prevalence (15.22%) of malnutrition in adult inpatients infected with SARS-CoV-2 in tertiary care centers across the US.

Malnutrition is a well-established independent predictor of adverse health outcomes and economic consequences, impacting patient recovery and healthcare costs [21,22]. In SARS-CoV-2 infection, malnourished individuals may experience further compromise by an uncontrolled surge in proinflammatory mediators that increase caloric expenditure and essential micronutrient requirements that compromise immunity and amplify infectivity. SARS-CoV-2 infection is often complicated by gut microbial dysbiosis that results in gastrointestinal dysfunction, curtailed nutrient intake, and even promotes malabsorption of nutrients [23]. In the present study, of 37,215 consecutive inpatients, 6807 (18.29%) reported diarrhea, 4503 (13.04%) reported poor appetite, and 2481 (7.18%) reported unintentional weight loss. Compromised nutrient intake, combined with an inflammation-driven catabolic state, leads to depletion of visceral proteins, protein-calorie malnutrition, and immunocompromise. These factors significantly increase the risk of viral virulence and adverse outcomes, including mortality [24].

We observed unique ordering patterns of ONS during the COVID-19 pandemic that are worth mentioning. Of the 5664 inpatients with malnutrition, irrespective of context or severity, 46.5% had no ONS ordered, while 53.5% had ONS ordered (Table 2). The mean age at admission was higher in patients given ONS than in those not given, while the median BMI of patients receiving ONS was lower than for those without ONS orders. The median length of stay in the ICU was longer for those receiving ONS than those who did not. The median length of stay in the hospital was longer for those receiving ONS than those without. This observation is consistent with our prior report that malnourished patients with COVID-19 illness tend to have more extended hospital and ICU stays when adjusting for comorbidities [14]. When compared to our institution’s historical ONS ordering over a one-year period (4747 of 153,161; 3.1% received ONS orders) [25], we observed that 7475 of 37,215 (20.09%) inpatients received ONS during the COVID-19 pandemic. Further, malnutrition patients who received ONS as inpatients were more likely to receive orders for ONS at discharge, 9.47% vs. 4.74% Table 2. As the COVID-19 pandemic progressed from pre-vaccine to vaccine phases, the ordering of ONS in patients with malnutrition varied. The highest frequency, 29.42%, occurred in the post-Omicron phase (Table 2), possibly reflecting the current greater awareness of the importance of nutritional intervention for inpatients with malnutrition, although a trend test did not confirm uniform progression across the five pandemic phases. Another possibility is that during the initial surge of COVID-19 physical barriers of patient isolation limited proper nutrition assessment. Ramos et al. [26] implemented a patient-generated electronic-based screening tool for risk screening to circumvent the issue of quarantine. Viñas et al. also reported variations in the prevalence of malnutrition according to the COVID-19 wave phenotype. They speculated that higher prevalences were reported in the pre-vaccine phase of the pandemic due to higher severity of illnesses [27].

Our previous report indicated that the mortality rate was significantly higher among malnourished inpatients (102 out of 403; 25.31%) compared to those who were not malnourished (453 out of 3908; 11.59%, *p* < 0.0001). Furthermore, malnourished inpatients were 76% more likely to experience mortality than their non-malnourished counterparts. [14]. In the present study, we extended our original observations to confirm that the inpatient mortality rate was higher in malnourished inpatients (740/5664; 13.06%) than those who were not malnourished (1472/31,551; 4.67%, *p* < 0.0001). Furthermore, malnourished inpatients had a greater likelihood of mortality compared to those who were not malnourished (OR = 3.07, 95% CI 2.80–3.37, *p* < 0.0001).

Thus, the primary objective of the present study was to determine if there was a survival advantage for hospitalized patients with malnutrition who received oral nutrition supplementation (ONS). Table 3 illustrates the multivariable logistic regression analysis reporting the possible association(s) between ONS, inpatient mortality, and malnutrition severity and context. After adjusting for potential covariates, patients with moderate and/or severe malnutrition in the context of acute illness or injury who were given ONS have lower odds of inpatient mortality in the weighted model (*p* < 0.001). In the adjusted model, patients with moderate malnutrition in the context of acute illness or injury who were given ONS were 28% less likely to have inpatient mortality (*p* < 0.0001). Furthermore, in our study, we observed that malnourished patients with severe malnutrition were more likely to receive ONS than those with moderate malnutrition (*p* = 0.02, Table 2). Given that patients with SARS-CoV-2 infection are being admitted with an acute injury-inflammatory subtype of malnutrition, this may provide an overlooked population to target with early screening and enteral nutrition.

Both higher and lower weight classes have been independently associated with a higher risk of mortality in COVID-19 [28,29]. Higher body mass indices (BMIs) have been independently associated with increased mortality among COVID-19 patients in critical care settings [18,30]. Thus, in the present study, we examined whether inpatient mortality in SARS-CoV-2-infected patients with malnutrition in ONS recipients varied by their weight class. The results of our analyses are shown in Table 4. In the weighted and adjusted models, overweight and obese patients who received ONS had higher odds of inpatient mortality (*p* < 0.0001, *p* = 0.02, respectively). This observation may be consistent with prior reports leading to American Society of Parenteral and Enteral Nutrition (ASPEN) guidelines advising to avoid overfeeding obese patients in the critical care setting [31]. In unadjusted analysis, ONS did not significantly affect inpatient mortality for those who were underweight (*p* = 0.0783), however after adjusting for covariates, ONS was protective of inpatient mortality in the weighted model (OR = 0.749, *p* < 0.001) and the adjusted model (OR = 0.776, *p* = 0.0001). Thus, patients who are malnourished or underweight appear to have a survival advantage when receiving ONS, while those who are overweight or obese are at higher risk of mortality. We then considered whether the timing of ONS reception in hospitalized patients with SARS-CoV-2 influenced mortality (Table 5). In the adjusted model, patients who had an earlier time to ONS had 13% lower odds of inpatient mortality (*p* = 0.0105). Earlier time to ONS after hospital admission has been shown to improve health economic outcomes. We previously reported that a 50% reduction in the time from hospital admission to initiation of oral nutritional supplements (ONS) was associated with a 10.2% overall decrease in length of stay (LOS) (*p* < 0.01) [25].

Malnutrition remains a significant contributor to poor health and economic outcomes in hospitalized patients. Individuals with malnutrition are at higher risk for prolonged hospitalizations, delayed recovery due to impaired wound healing, increased susceptibility to infections and medical complications, and heightened rates of morbidity and mortality [32]. As a result, malnourished patients tend to have greater care needs and utilize more hospital resources, which contributes to increased healthcare expenditures [33]. Early recognition and nutrition-based interventions of adult malnourished inpatients improve health-economic outcomes, including mortality [25,34]. Both ASPEN and the European Society for Parenteral and Enteral Nutrition (ESPEN), along with the Centers for Medicare & Medicaid Services (CMS) and the Joint Commission, recommend early nutrition risk screening for adult inpatients, as well as prompt nutrition-based interventions for those identified with or at risk of malnutrition [12,35,36].

Supporting the importance of targeted nutrition interventions, a double-blind, placebo-controlled clinical trial found that high-protein, hydroxy-methyl-butyrate-enriched ONS significantly reduced 90-day mortality among hospitalized, malnourished elderly individuals with chronic comorbidities such as COPD, heart failure, acute myocardial infarction, or pneumonia, compared with placebo (4.8% vs. 9.7%; relative risk 0.49, 95% CI 0.27–0.90; *p* = 0.018) [13,37]. Kaegi-Braun et al. analyzed a cohort of 114,264 hospitalizations of inpatients with malnutrition in a nationwide Swiss claims database to determine the association of hospital mortality with the delivery of ONS [38]. In a matched cohort of 34,967 inpatients with confirmed malnutrition (30.6%), those who received nutritional support had a lower in-hospital mortality rate compared to those who did not (7.2% [2525/34,967] vs. 8.8% [3072/34,967]; IRR, 0.79; 95% CI, 0.75–0.84; *p*  <  0.001), as determined by 1:1 propensity score matching. Scheutz et al. conducted a pragmatic open-labeled clinical trial of adult hospitalized inpatients at 8 Swiss hospitals. Of 5015 patients screened, 2088 were recruited, with 1050 patients assigned to the intervention group and 1038 to the control group. In a randomized controlled trial of inpatients identified at nutritional risk using the Nutrition Risk Screening-2002 tool, participants assigned to protocol-guided individualized nutritional support targeting protein and caloric goals had significantly lower 30-day mortality (7% [73/1043]) compared to those receiving standard hospital food (10% [100/1047]; adjusted OR, 0.65; 95% CI, 0.47–0.91; *p* = 0.011) [39]. 

Gomes et al. conducted a meta-analysis of clinical trials of non-critically ill adult inpatients with malnutrition who underwent a nutrition-based intervention with oral or enteral nutrition vs. usual care, with the primary endpoint of mortality [40]. A meta-analysis of 27 randomized trials (n = 6803) found that nutritional support significantly reduced mortality compared with control (8.3% [230/2758] vs. 11.0% [307/2787]; OR 0.73; 95% CI, 0.56–0.97). Sensitivity analyses indicated a greater mortality reduction in recent trials (2015 or later, OR 0.47; 95% CI, 0.28–0.79), among patients with established malnutrition (OR 0.52; 95% CI, 0.34–0.80), and in studies with high protocol adherence (OR 0.67; 95% CI, 0.54–0.84). Kaegi-Braun et al. from this group updated the meta-analysis in 2021 to include 7166 inpatients who underwent oral or enteral nutrition vs. usual care [41]. A total of 20 studies reported data on the primary outcome of mortality showing that nutritional support leads to a significant 28% reduction in mortality among hospitalized patients, with an odds ratio of 0.72 (95% CI, 0.57–0.91; *p* = 0.006). Importantly, subgroup analyses highlight that high-protein supplementation and longer duration of nutritional intervention are the most influential factors predicting improved survival, as reflected by the significantly lower odds ratios and statistical heterogeneity in these groups.

Clinical evidence regarding oral or enteral nutrition in hospitalized COVID-19 patients is sparse [42]. However, preliminary findings from a retrospective study suggest that early enteral nutrition could improve outcomes, as evidenced by reduced hospital stay, fewer respiratory complications, and less frequent adverse events, though these results are based on a small, underpowered cohort. Among 75 hospitalized COVID-19 patients, only 21 received nutritional support and just 12 received early enteral nutrition [43].

Chen et al. conducted a single-center cohort study from a limited dataset of 1181 patients hospitalized at Shanghai Fourth People’s Hospital with symptomatic COVID-19, aged 65 or older, during the Omicron wave of the pandemic from April to June 2022 [44]. Overall, they reported that the mortality was 1.2% in those receiving ONS (n = 258) compared to 4.3% in the non-ONS group (n = 258). However, early delivery of ONS (<48 h, n = 120 patients) vs. late delivery (>48 h, n = 120 patients) was not associated with improved mortality. In our study, we observed a 13% survival advantage in those receiving early vs. late oral nutrition supplementation. Chen et al. also noted significant study limitations, including a small sample size that was imbalanced between the groups, which may affect the generalizability of the study results. Another key limitation of this study is that malnutrition prevalence was neither reported nor considered when determining the provision of nutritional support, potentially confounding the interpretation of nutritional intervention outcomes.

Recognizing that malnutrition is an independent risk factor for longer hospitalizations and higher mortality [45], integrating early enteral nutrition management strategies warrants consideration as a standard of care, so long as their implementation is grounded in robust scientific methodology [11,46].

As with most studies, our study has limitations that should be considered when interpreting the findings. Although our sample size was robust, the prevalence of malnutrition was insufficient to allow for analysis within all subsets of malnourished patients, most notably malnutrition in the context of social or environmental circumstances and starvation. This excludes analyses for an essential population of homeless or impoverished persons who were at higher risk of adverse clinical outcomes with SARS-CoV-2 infection.

Although our cohort included patients from five hospitals within a large regional health system, the findings may not necessarily extend to populations outside the Baltimore–Washington, DC area. In addition, unmeasured confounding inherent to observational designs remains a consideration. Our cohort study lacks important information on the quantity and duration of ONS consumed relative to health economic outcomes. Ideally, a double-blind placebo-controlled clinical study that randomizes ONS to a placebo drink in SARS-CoV-2-infected inpatients would be ideally suited to determine if ONS influences the survival of SARS-CoV-2-infected adult inpatients.

## 5. Conclusions

Malnutrition is an independent predictor of higher hospital mortality from COVID-19 illness. We report the most extensive collection of 37,215 consecutive inpatients evaluated for malnutrition during the 5 phases of the COVID-19 pandemic. We observed a 15.22% prevalence of malnutrition in adult inpatients infected with SARS-CoV-2. Inpatient adults with moderate and/or severe malnutrition in the context of acute illness or injury who were given ONS had significantly lower odds of inpatient mortality than patients who did not receive ONS. In contrast, overweight and obese patients who received ONS had significantly higher odds of inpatient mortality. ONS was significantly protective against inpatient mortality. Thus, patients who were malnourished or underweight appeared to have a hospital survival advantage when receiving ONS, while those who were overweight or obese were at higher risk of mortality. Patients who had an earlier time to ONS had significantly lower odds of inpatient mortality.

Our findings reinforce that malnutrition is a key determinant of mortality among COVID-19 inpatients. There is a clear need for rigorously designed interventional studies exploring the effects of prompt nutritional assessment and oral supplementation, particularly targeting adult hospitalized individuals. Implementing such interventions early in the course of hospitalization may contribute meaningfully to improving patient prognosis.

## Figures and Tables

**Table 1:** Summary of COVID-19 inpatients from 1 March 2020 to 31 March 2023; N = 37,215.

Characteristic	Mean (SD) or Median (IQR)
Age (years)	57.56 (20.2)
Body mass index (kg/m^2^)	28.3 (24.3, 33.7)
Length of Stay in ICU (days)	5.2 (2.8, 10.3)
Length of Stay in Hospital (days)	4.1 (2.4, 7.6)
	**N (%)**
**Gender**	
Male	15,117 (40.62)
Female	22,092 (59.36)
Non-binary	2 (0.01)
Unknown	4 (0.01)
**Race**	
White	18,487 (49.68)
Black	12,844 (34.51)
Asian	1710 (4.59)
Other	4174 (11.22)
**Insurance Group**	
Commercial	18,830 (50.60)
Government	12,010 (32.27)
Self-pay	1644 (4.42)
State-run	2913 (7.83)
Others	1816 (4.88)
**Admission Source**	
Home, workplace, or non-healthcare facility; court/law enforcement	30,496 (82.05)
Physician’s office or clinic; other healthcare facility	2696 (7.25)
Skilled nursing facility, intermediate care facility, or assisted living facility	2064 (5.55)
Transfers from another acute care hospital or ED	1910 (5.14)
**Characteristic**	N (%)
**Weight Category**	
Normal	8367 (25.64)
Obese	13,533 (41.47)
Overweight	9682 (29.67)
Underweight	1049 (3.21)
**Comorbidity**	
Diabetes	12,579 (33.8)
Hypertension	24,015 (64.53)
Diarrhea	6807 (18.29)
COPD	2963 (7.96)
Malnutrition by ICD-10	5664 (15.22)
**Malnutrition Severity**	
Moderate	1861 (51.64)
Severe	1743 (48.36)
**Malnutrition Context**	
Acute disease or injury	1439 (39.66)
Chronic disease or condition	1813 (49.97)
Social or environmental circumstances, starvation	352 (9.7)
Other	24 (0.66)
**Poor Appetite**	4668 (13.55)
**Unintentional Weight Loss**	2508 (7.28)
**ONS Ordered**	
Yes	7475 (20.09)
No	29,740 (79.91)
**Discharge ONS**	
Yes	843 (2.27)
No	36,372 (97.73)
**COVID Wave**	
1 Pre-Vaccine	11,333 (30.45)
2 Pre-Delta	5552 (14.92)
3 Delta	5033 (13.52)
4 Omicron	5620 (15.1)
5 Post-Omicron	9677 (26)
**Readmission**	6763 (18.17)
**ICU**	11,345 (30.49)
**Inpatient Mortality**	
Deceased	2212 (5.94)
Discharged	35,003 (94.06)

**Table 2:** Characteristics of COVID-19 inpatients with malnutrition stratified by ONS, who were admitted to a JHH-affiliated hospital from 1 March 2020 to 31 March 2023 (n = 5664).

	No ONS Ordered	ONS Ordered	
	N = 2635; 46.5%	N = 3029; 53.5%	
**Characteristic**	**Mean (SD)** or Median (IQR)	**Mean (SD)** or Median (IQR)	***p*-value**
Age (years)	61.77 (19.5)	67.17 (16.9)	<0.0001
Body mass index (kg/m^2^)	24.9 (20.8, 31.6)	23.2 (19.9, 27.5)	<0.0001 *
Length of Stay in ICU (days)	5.3 (2.7, 10.9)	12.1 (6.9, 22.7)	<0.0001 *
Length of Stay in Hospital (days)	4.6 (2.4, 8.9)	11.1 (6.6, 20.8)	<0.0001 *
	**No ONS Ordered** **N (%)**	**ONS Ordered** **N (%)**	***p*-value**
Male	1244 (47.21)	1526 (50.38)	0.02
Female	1391 (52.79)	1503 (49.62)	
**Race**			
White	1320 (50.09)	1528 (50.45)	0.52
Black	1022 (38.79)	1191 (39.32)	
Asian	94 (3.57)	112 (3.7)	
Other	199 (7.55)	198 (6.54)	
**Insurance Group**			
Commercial	1088 (41.29)	1143 (37.74)	<0.0001
Government	1094 (41.52)	1498 (49.46)	
Self-pay	91 (3.45)	61 (2.01)	
State-run	236 (8.96)	222 (7.33)	
Others	126 (4.78)	105 (3.47)	
**Weight Category**			
Normal	869 (38.74)	1215 (45.64)	<0.0001
Obese	665 (29.65)	435 (16.34)	
Overweight	450 (20.06)	599 (22.5)	
Underweight	259 (11.55)	413 (15.51)	
**Characteristic**	**No ONS Ordered** **N (%)**	**ONS Ordered** **N (%)**	***p*-value**
**Admission Source**			
Home, workplace, or non-healthcare facility; court/law enforcement	2063 (78.47)	2108 (69.64)	<0.0001
Physician’s office or clinic; other healthcare facility	178 (6.77)	285 (9.42)	
Skilled nursing facility, intermediate care facility, or assisted living facility	225 (8.56)	350 (11.56)	
Transfers from another acute care hospital or ED	163 (6.2)	284 (9.38)	
Diabetes	1108 (42.05)	1299 (42.89)	0.53
Hypertension	1996 (75.75)	2452 (80.95)	<0.0001
Diarrhea	858 (32.56)	1027 (33.91)	0.28
COPD	381 (14.46)	394 (13.01)	0.11
**Malnutrition Severity**			
Moderate	693 (53.97)	1120 (49.91)	0.02
Severe	591 (46.03)	1124 (50.09)	
**Malnutrition Context**			
1 (Acute disease or injury)	528 (40.52)	873 (38.89)	<0.0001
2 (Chronic disease or condition)	622 (47.74)	1161 (51.71)	
3 (Social or environmental circumstances, starvation)	134 (10.28)	210 (9.35)	
Other	19 (1.46)	1 (0.04)	
**Poor Appetite**	571 (22.96)	932 (31.98)	<0.0001
**Unintentional Weight Loss**	415 (16.69)	670 (22.99)	<0.0001
**Discharge ONS**	125 (4.74)	295 (9.74)	<0.0001
**COVID Wave**			
1 Pre-Vaccine	738 (28.01)	717 (23.67)	<0.05
2 Pre-Delta	344 (13.06)	377 (12.45)	
3 Delta	337 (12.79)	476 (15.71)	
4 Omicron	456 (17.31)	568 (18.75)	
5 Post-Omicron	760 (28.84)	891 (29.42)	
**Readmitted**	931 (35.33)	1389 (45.86)	<0.0001
**ICU**	1187 (45.05)	982 (32.42)	<0.0001
**Inpatient mortality**	271 (10.28)	469 (15.48)	<0.0001

* *t*-test on log-transformed variables.

**Table 3:** Multivariable logistic regression models for association between ONS and inpatient mortality stratified by malnutrition severity and context.

	Moderate Acute Illness or Injury	Moderate Chronic Illness	Severe Acute Illness or Injury	Severe Chronic Illness
Total Patients	676	966	763	847
Deceased [N (%)]				
No ONS	24 (9.38)	25 (6.65)	43 (14.58)	32 (11.99)
Had ONS	51(12.14)	101 (17.12)	71 (15.17)	111 (19.14)
*p*-value	0.27	<0.0001	0.82	0.01
Base ^1^				
Odds Ratio	1.34	2.9	1.05	1.74
95% CI	0.8–2.23	1.83–4.59	0.7–1.58	1.14–2.65
*p*-value	0.27	<0.0001	0.82	0.01
Weighted ^2^				
Odds Ratio	0.67	1.36	0.74	1.63
95% CI	0.57–0.78	1.19–1.57	0.65–0.84	1.42–1.87
*p*-value	<0.0001	<0.0001	<0.0001	<0.0001
Adjusted ^3^				
Odds Ratio	0.72	1.4	0.76	1.37
95% CI	0.62–0.85	1.21–1.62	0.67–0.87	1.18–1.58
*p*-value	<0.0001	<0.0001	<0.0001	<0.0001

^1^ The base model is unadjusted and unweighted. ^2^ Weighted model adjusts for inverse probability weights derived from modeling for receipt of ONS incorporating age, gender, race, BMI, malnutrition diagnosis, date of admission, diabetes, hypertension, diarrhea, COPD, poor appetite, unexplained weight loss, admission source, hospital length of stay and ICU admission in the entire cohort. ^3^ The adjusted model additionally adjusts for the COVID wave and insurance group.

**Table 4:** Multivariable linear regression models for association between ONS and inpatient mortality stratified by BMI group.

	Normal	Obese	Overweight	Underweight
Total Patients	8367	13,502	9682	1043
**Deceased** [N (%)]				
No ONS	314 (5.26)	340 (2.91)	302 (3.82)	46 (8.41)
Had ONS	285 (11.88)	182 (10.03)	217 (12.23)	58 (11.69)
*p*-value	<0.0001	<0.0001	<0.0001	0.0771
**Base ^1^**				
Odds Ratio	**2.43**	**3.72**	**3.51**	**1.44**
95% CI	2.05–2.87	3.09–4.49	2.92–4.21	0.96–2.17
*p*-value	<0.0001	<0.0001	<0.0001	0.0783
**Weighted ^2^**				
Odds Ratio	0.95	1.11	1.16	0.75
95% CI	090–0.999	1.04–1.18	1.09–1.24	0.66–0.85
*p*-value	0.0477	0.0008	<0.0001	<0.0001
**Adjusted ^3^**				
Odds Ratio	0.96	1.08	1.15	0.78
95% CI	0.91–1.01	1.01–1.14	1.08–1.22	0.68–0.88
*p*-value	0.1148	0.0216	<0.0001	0.0001

^1^ The base model is an unadjusted, unweighted model using the original raw data. ^2^ The weighted model incorporates weights for receipt of ONS. ^3^ The adjusted model incorporates weights for receipt of ONS and adjustment for COVID wave and insurance group.

**Table 5:** Multivariable Linear Regression Models for Association Between Time from Hospital Admission to ONS and Inpatient Mortality (n = 7475).

	Patients with ONS Ordered
Total Patients	7475
**Time from Hospital Admission** **to ONS in days [Median (IQR)]**	
Discharged Alive (N = 6617)	4.12 (2.32, 7.67)
Deceased in Hospital (N = 858)	4.16 (2.45, 8.09)
*p*-value	0.1035 ^1^
**Adjusted ^2^**	
Odds Ratio	0.87
95% CI	0.79–0.97
*p*-value	0.0105 ^2^

^1^ Regression analysis: Time from hospital admission to ONS order in days and length of stay in hospital were log-transformed. ^2^ The adjusted model includes age, gender, race, BMI, malnutrition diagnosis, diabetes, hypertension, diarrhea, COPD, poor appetite, unexplained weight loss, admission source, hospital length of stay, ICU admission, COVID wave, and insurance group as covariates.

## Data Availability

Data used in this publication are part of JH-CROWN: The COVID-19 PMAP Registry [15]. Deidentified data may be made available pursuant to a data use agreement and appropriate Johns Hopkins Institutional Review Board approval (https://www.hopkinsmedicine.org/institutional_review_board/news/covid19_information/index.html accessed on 26 June 2023). Analytical code is available from the corresponding author upon reasonable request.

## Appendix A

**Table A1:** Summary of inpatients by ONS order status.

	No ONS Ordered	ONS Ordered	
	N = 29,740	N = 7475	
**Characteristic**	**Mean (SD)** or Median (IQR)	**Mean (SD)** or Median (IQR)	***p*-value**
Age (years)	55.31 (20.1)	66.5 (17.5)	<0.0001
Body mass index (kg/m^2^)	29 (25, 34.3)	25.8 (21.9, 30.7)	<0.0001 *
Length of Stay in ICU (days)	4.1 (2.3, 7.3)	10.8 (6.3, 18.1)	<0.0001 *
Length of Stay in Hospital (days)	3.3 (2.2, 5.5)	10.2 (6.2, 17.5)	<0.0001 *
	**N (%)**	**N (%)**	***p*-value**
Gender			
Male	11,504 (38.69)	3613 (48.33)	<0.0001
Female	18,230 (61.31)	3862 (51.67)	
**Race**			
White	14,754 (49.61)	3733 (49.94)	<0.0001
Black	10,111 (34)	2733 (36.56)	
Asian	1419 (4.77)	291 (3.89)	
Other	3456 (11.62)	718 (9.61)	
**Insurance Group**			
Commercial	15,924 (53.55)	2906 (38.88)	<0.0001
Government	8522 (28.66)	3488 (46.66)	
Self-pay	1390 (4.67)	254 (3.4)	
State-run	2412 (8.11)	501 (6.7)	
Others	1490 (5.01)	326 (4.36)	
**Weight Category**			
Normal	5967 (22.83)	2400 (36.97)	<0.0001
Obese	11,714 (44.81)	1819 (28.02)	
Overweight	7907 (30.25)	1775 (27.35)	
Underweight	552 (2.11)	497 (7.66)	
**Admission Source**			
Home, workplace, or non-healthcare facility; court/law enforcement	25,005 (84.21)	5491 (73.49)	<0.0001
Physician’s office or clinic; other healthcare facility	2131 (7.18)	565 (7.56)	
Skilled nursing facility, intermediate care facility, or assisted living facility	1261 (4.25)	803 (10.75)	
Transfers from another acute care hospital or ED	1297 (4.37)	613 (8.2)	
**Characteristic**	**No ONS Ordered** **N (%)**	**ONS Ordered** **N (%)**	***p*-value**
**Comorbidity**			
Diabetes	9304 (31.28)	3275 (43.81)	<0.0001
Hypertension	18,071 (60.76)	5944 (79.52)	<0.0001
Diarrhea	4799 (16.14)	2008 (26.86)	<0.0001
COPD	2062 (6.93)	901 (12.05)	<0.0001
Malnutrition by ICD-10	2635 (8.86)	3029 (40.52)	<0.0001
**Malnutrition Severity**			
Moderate	722 (54.2)	1139 (50.13)	0.02
Severe	610 (45.8)	1133 (49.87)	
**Malnutrition Context**			
1 (Acute disease or injury)	551 (40.66)	888 (39.07)	<0.0001
2 (Chronic disease or condition)	643 (47.45)	1170 (51.47)	
3 (Social or environmental circumstances, starvation)	138 (10.18)	214 (9.41)	
Other	23 (1.7)	1 (0.04)	
**Poor Appetite**	2484 (9.1)	2184 (30.49)	<0.0001
**Unintentional Weight Loss**	1418 (5.2)	1090 (15.22)	<0.0001
**Discharge ONS**	328 (1.1)	515 (6.89)	<0.0001
**COVID Wave**			
1 Pre-Vaccine	9056 (30.45)	2277 (30.46)	<0.01
2 Pre-Delta	4433 (14.91)	1119 (14.97)	
3 Delta	4068 (13.68)	965 (12.91)	
4 Omicron	4394 (14.77)	1226 (16.4)	
5 Post-Omicron	7789 (26.19)	1888 (25.26)	
**Readmitted**	4236 (14.24)	2527 (33.81)	<0.0001
**ICU**	8376 (28.16)	2969 (39.72)	<0.0001
**Inpatient mortality**	1354 (4.55)	858 (11.48)	<0.0001

* *t*-test on log-transformed variable.
